# Statins use amidst the pandemic: prescribing, dispensing, adherence, persistence, and correlation with COVID-19 statistics in nationwide real-world data from Poland

**DOI:** 10.3389/fphar.2024.1350717

**Published:** 2024-04-09

**Authors:** Przemysław Kardas, Angelika Kwiatek, Piotr Włodarczyk, Filip Urbański, Beata Ciabiada-Bryła

**Affiliations:** ^1^ Medication Adherence Research Centre, Department of Family Medicine, Medical University of Lodz, Łódź, Poland; ^2^ e-Health Centre, Warsaw, Poland; ^3^ Department of Preventive Medicine, Faculty of Health Sciences, Medical University of Lodz, Łódź, Poland

**Keywords:** statins, COVID-19 pandemic, adherence, persistence, prescribing, dispensation, Poland, real-word data

## Abstract

**Background:**

Adherence to medications presents a significant challenge in healthcare. Statins, used in primary and secondary prevention of cardiovascular disease, are of particular importance for public health. The outbreak of the COVID-19 pandemic resulted in additional healthcare system-related barriers impeding the execution of therapies. This study aimed to assess the use of as well as adherence and persistence to statins in a national cohort of 38 million of Polish citizens during pandemic.

**Methods:**

A retrospective analysis of prescription and dispensation data for all statins users from the national payer organization covering the years 2020–2022 was conducted. Medication adherence was assessed using the Medication Possession Ratio, for persistence the 30-day cut-off was accepted. National data on COVID-19 cases and COVID-19 related deaths were obtained from ECDC.

**Results:**

The analysis identified 7,189,716 Polish citizens (approximately 19% of Polish population) who were dispensed at least 1 pack of statins within the study period. Over that time, there was a continuous significant increasing trend in prescribing and dispensing of statins. Despite a total increase of 18.9% in the number of prescribed tablets, the percentage of tablets dispensed remained similar, averaging 86%. Overall percentage of adherent patients was 48.2%. For a random sample of 100,000 patients, the mean period of continuous therapy in 2022 was 6.2+/- 5.3 months. During the lockdown period, the mean number of prescribed and dispensed tablets was lower by 6.8% and 5.9%, respectively (*p* < 0.05). However, fluctuations in the number of COVID-19 cases or COVID-19-related deaths per week had no major impact on the prescribing and dispensing of statins.

**Conclusion:**

Over the time of pandemic, there was a continuous increase in the number of statin tablets prescribed and dispensed in Poland. This suggests that, despite the potential limitations posed by COVID-19, access to statins remained easy, which may be attributed to the mass-scale implementation of the national e-prescription system. However, it is crucial to realise that approximately 1/7 of prescribed statin doses were never dispensed, and the overall levels of adherence and persistence were low. This underscores the necessity for concerted efforts to change this scenario in Poland.

## 1 Introduction

Despite the outstanding progress medicine has made in the last decades, cardiovascular diseases still account for a large portion of morbidity and mortality. In Poland, over 40% of total deaths are attributed to cardiovascular disease, with atherosclerotic cardiovascular disease (ASCVD) ranking as the leading cause of mortality (Główny Urząd Statystyczny, 2020). Various forms of hyperlipidaemias, and particularly those leading to elevated levels of low-density lipoprotein cholesterol (LDL-C), are considered a significant causal risk factor for the development of ASCVD. Therefore, the key to primary and secondary prevention is to decrease LDL-C levels by lipid-lowering therapies. The currently applicable European as well as Polish guidelines on dyslipidaemia management recommend long-term, usually lifelong therapies with lipid-lowering drugs in order to reduce the cardiovascular risk (Mach et al., 2020; Szymański et al., 2022). Despite new drugs that have emerged over the last years, statins play a cardinal role in effective management of this problem, owing to the extensive evidence of their effectiveness in both primary and secondary prevention (Author Anonymous, 1994; Collins et al., 2003). Consequently, they are of absolutely fundamental importance to public health, representing one of the most often prescribed groups of drugs (De Vera et al., 2014; Fuentes et al., 2018).

Unfortunately, the effectiveness of statins is negatively affected by suboptimal execution of therapy in real-life settings. Patients fail to adhere to the treatment in different ways, corresponding with all three phases of adherence as defined by the ABC terminology (Vrijens et al., 2012). Namely, they 1) do not initiate the treatment (which holds the name of “primary non-adherence”); 2) poorly execute their daily therapy (which typically is referred to as “poor adherence”, however, according to ABC taxonomy, should be rather called “poor implementation”); and 3) discontinue the therapy, revealing poor persistence.

All these ways of behaviour have negative consequences. Non-adherence and non-persistence with statins lead to ineffectiveness in LDL-C level reduction in both primary and secondary prevention (Shalev et al., 2014). Consequently, they significantly elevate the risk of fatal and nonfatal cardiovascular events, as well as mortality. A systematic review of studies indicates an increased CVD risk ranging from 1.22 to 5.26, and mortality risk ranging from 1.25 to 2.54 among non-adherent individuals. Non-persistent individuals, on the other hand, face an elevated risk of CVD ranging from 1.22 to 1.67, and mortality risk ranging from 1.79 to 5.00 (De Vera et al., 2014). Non-adherence and non-persistence with statin therapy also has an impact on hospitalization costs and other CVD-related costs (Bansilal et al., 2016).

Of importance is the fact that there are some objective thresholds that allow for obtaining a full benefit of statin therapy. Namely, the most consistent benefits were observed at an adherence level of at least 80%. In primary prevention cohorts, clinical benefits usually occurred after 1 year of continuous therapy, whereas longer duration of treatments were associated with additional improvements in outcomes (Simpson and Mendys, 2010; Deshpande et al., 2017).

Medication adherence is affected by a number of factors. A useful model created by the World Health Organization (WHO) categorizes them into five distinct clusters that encompass health system, therapy, condition, patient, and socioeconomic factors World Health Organization (2003). The last few years were characterised by a strong effect of an element that penetrated to several of these clusters, namely, the COVID-19 pandemic. Not only did it exert a major impact on morbidity and mortality (accounting for nearly 18% of total deaths in Poland in 2021 (Statistics Poland, 2021)), but it also had both direct and indirect effect on adherence, seriously limiting access to healthcare services. Additionally, it negatively affected economies and daily life of citizens due to lockdowns, social distancing, remote mode of work, etc. (Ágh et al., 2021; Kardas et al., 2021; Agh et al., 2023).

Medication adherence can be assessed using a variety of measurement techniques. For instance, the implementation phase can be evaluated through dedicated questionnaires, urine and blood tests to detect drug presence, or electronic drug monitors. In the case of persistence and discontinuation, pharmacy claims data and insurers’ databases are commonly utilized. Of course, each of these methods has its limitations. However, depending on the specific aspect of interest, many of them are employed in current research. Unfortunately, their utilization in clinical practice is much less common. As a result, in many countries, including Poland, adherence is not systematically monitored (Kardas, 2024).

Interestingly, just before the outbreak of COVID-19 pandemic, the e-prescription system was introduced on the mass scale in Poland. Polish e-prescriptions contain details of patient characteristics such as name, date of birth, and national identification number (but not clinical indications), as well as the qualitative and quantitative characteristics of prescribed drugs. Once authorized by the prescriber, they are stored in a centralized database, where their status changes in case of partial or complete dispensation. Therefore, this allowed for comparison of prescribing and dispensing data in an unprecedentedly precise way. Taking advantage of this opportunity, this study was aimed to examine the use of statins in Poland during the pandemic, with the particular focus on exploring the prescribing and dispensing patterns, medication adherence, persistence, and their relationship with COVID-19 statistics.

## 2 Methods

This retrospective analysis was based on data from e-prescriptions issued and dispensed in Poland between 2020 and 2022. As the nationwide e-prescription system was launched on 8 January 2020, the analysis period for prescription and dispensation was confined to 8 January 2020 to 31 December 2022, allowing access to the complete dataset.

### 2.1 Source data

The data used in this study was retrieved from the databases of e-Health Centre (Polish: Centrum e-Zdrowia), a governmental institution responsible for managing the Polish national eHealth system, including a nationwide e-prescription system. Each individual record contained information on the date of prescription, details of the prescribed drug (such as the trade name, dose, number of packs, etc.), date of dispensation (if it took place), as well as details of the drug dispensed. The basic patient characteristics (i.e., age and sex) were also recorded, however, no clinical data were collected or analysed. For the analysis of the cohort, patients were included with their first statin dispensation. Original prescription and dispensation data were presented with weekly granularity, and expressed in units of tablets. The percentage of tablets dispensed was also calculated in a similar way. When applicable, the data were further recalculated using a 30-tablet pack as the standard unit.

The primary focus of this analysis were statins, i.e., lipid-modifying agents being HMG CoA reductase inhibitors. It included both drugs formed from a single chemical compound, as well as fixed-dose combination of lipid-lowering medications, whereas fixed-dose drugs containing statins and non-lipid-lowering drugs were not taken into consideration. Other lipid-lowering drugs, such as ezetimibe or fibrates, were not considered.

Consequently, the analysis covered patients fulfilling the following inclusion criteria.

1. Patients prescribed drugs corresponding with one of the codes of the Anatomical Therapeutic Chemical (ATC) classification provided below:
a) statins (ATC code: C10AA)
b) fixed combinations of statins and other lipid-modifying agents (ATC codes: C10BA01 - C10BA09, C10BA11 and C10BA12)
2. Patients dispensed at least one pack of such a drug within the analysed period.

No exclusion criteria were employed.

Of a note is that the Polish regulations support prescribing and dispensing of the drugs in original packs, as manufactured. However, packs differ in size, most often containing either 28, or 30 tablets in the pack, or multiplicity of these numbers. Adopting one tablet as the basic unit for this analysis allowed for avoiding a potential bias resulting from various sizes of packs. Moreover, Polish legislation permits generic substitution, and thus a drug specified in a prescription may be substituted by another drug with the same active compound and potency. Therefore, for the purpose of this analysis, all the drugs containing statins and corresponding to one of the above-listed ATC codes were considered as interchangeable, and the daily dose was one tablet, regardless of the compound and dosage prescribed.

The outbreak of the COVID-19 occurred in December 2019, whereas the first Polish COVID-19 case was reported on 4 March 2020. The World Health Organization announced the COVID-19 pandemic on 11 March 2020, and declared its end on 5 May 2023 (United Nations News, 2024). Hence, the timeframe included in this analysis, spanning from January 2020 to December 2022, was entirely impacted by the pandemic.

For the purpose of this analysis, national data on COVID-19 cases and COVID-19-related deaths were obtained from the publicly available databases of the European Centre for Disease Prevention and Control (ECDC), with weekly granularity applied (ECEC Data on the daily number of new reported COVID-19 cases and deaths by EU/EEA country, 2024).

During the COVID-19 pandemic in Poland, there were three lockdowns: the first one from 14 March to 31 May 2020, the second from 7 November to 28 December 2020, and the last one from 20 March to 26 April 2021. These periods correspond with weeks 12–22 and weeks 46–52 of the year 2020, as well as weeks 12–16 of the year 2021. Therefore, out of the 156 weeks studied, 23 were considered to be lockdown periods.

According to the national statistical office (Statistics Poland), the population of Poland (in thousands of inhabitants, as of December 31) was 38,089, 37,908 and 37,766 for years 2020, 2021 and 2022, respectively (Główny Urząd Statystyczny, 2020).

### 2.2 Adherence and persistence definitions

Adherence and persistence were computed for each patient over the study duration, being two key complementary parameters describing long-term drug taking.

Adherence was defined as the degree to which a patient adheres to the prescribed medication regimen. Medication adherence was assessed using the Medication Possession Ratio (MPR), which represents the proportion of days for which medication was supplied in relation to the total days of the intended treatment. Assuming that statin therapy is typically lifelong, the analysis period for each patient was defined from the day of dispensation of the first statin prescription until 31 December 2022. Patients with an MPR below 0.80 (indicating that they possessed medication for less than 80% of the prescribed treatment period) were classified as non-adherent.

Persistence, on the other hand, measured whether patients continued to refill their statin prescriptions during the analysis period. Non-dispensation was defined as a failure to collect a medication within 30 days after the supply dispensed based on the preceding e-prescription had run out. Consequently, non-persistence was deemed to occur when a patient refrained from refilling their statin medication for a period exceeding 30 days.

### 2.3 Statistical analysis

Statistical analysis involved descriptive statistics of both prescribing and dispensing, as well as the percentage of drugs dispensed (compared to drugs prescribed). All the data were expressed with weekly granularity. The Shapiro-Wilk W test was used to study the normal distribution of continuous variables. If the assumption of normality was met, then means were used in subsequent stages of the analysis. Otherwise, medians were used.

Categorical variables were expressed as proportions and compared between relevant groups using the χ2 test. To compare the average percentage of statin tablets dispensed for every out of three studied years, the Kruskal–Wallis test was used. The *p*-value of less than 0.05 was considered significant. Statistical calculations were made with the use of Statistica 13.1 software (TIBCO Software Inc.).

### 2.4 Ethical issues

The source data were fully anonymised. Similarly, all the findings were reported in an aggregated manner, and no individual data were disclosed. Therefore, in accordance with the policy of the Ethics Committee of the Medical University of Lodz, the study did not require ethical approval.

## 3 Results

### 3.1 General data

Throughout the entire analysed period, there were more and more new individuals who were dispensed statins, and thus satisfied the inclusion criterion. They were added to the analysed cohort with a steady mean pace of 2,794 per day. In total, between 1 January 2020 and 31 December 2022, as many as 7,189,716 Polish citizens (i.e., approximately 19% of the Polish population) were dispensed at least one pack of statins. Out of this number, 695,636 died in the analysed period. The mean age of these individuals ± standard deviation (SD), calculated on 31 December 2022 or the day of their death, was 67.6 ± 12.3 years. In the group discussed, 55.4% were females and 44.4% were males (gender data was missing for 0.2%).

### 3.2 Statins prescribed and dispensed

Between 8 January 2020 and 31 December 2022, 4,681,614,262 statin tablets were prescribed, and 4,010,716,658 dispensed in Poland. If recalculated as typical 30-tablet packs, these numbers translate into 156,053,809 (52,017,936 per year, on average) packs prescribed, and 133,690,555 (44,563,518 per year) packs dispensed.

**FIGURE 1:**
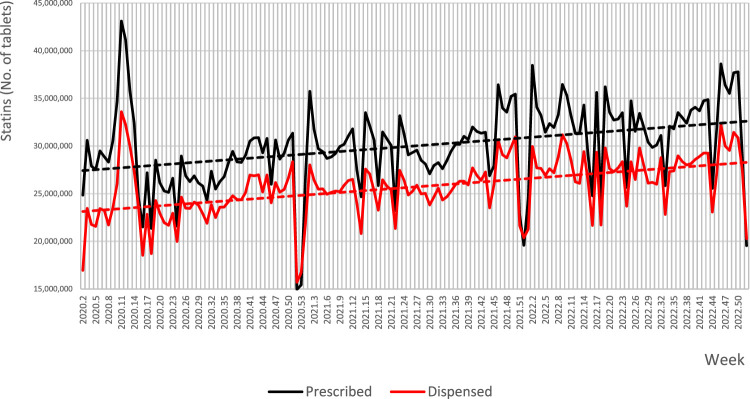
Weekly numbers and longitudinal trends of prescribed and dispensed statins in Poland from 8 January 2020 to 31 December 2022. Note: dotted lines represent trend lines.

**FIGURE 2:**
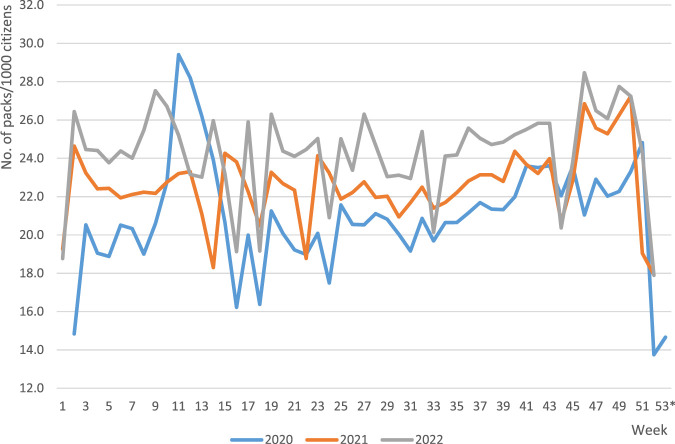
Annual trends of weekly number of statin packs dispensed per 1000 citizens of Poland from 8 January 2020 to 31 December 2022. Note: Standard 30-tablet packs were the basis for calculation. *week 53 applicable to year 2020 only.

The quantities of prescribed and dispensed statins were subject to weekly and seasonal variation, going in parallel for both these parameters. What is noteworthy is, e.g., a sharp drop in the quantity of prescribed and dispensed drugs within the last 2 weeks of each year, with a subsequent sharp rise in the first weeks of the following year. Nevertheless, a continuous rising trend was observed for both these parameters along the entire analysed period (Figure 1). Consequently, when studying the trends of these data, it may be observed that the weekly number of prescribed tablets of stains increased between 8 January 2020 and 31 December 2022 from 27,418,954 to 32,601,689, i.e., by 18.9%, and the number of dispensed tablets from 23,133,631 to 28,285,831, i.e., by 22.3%, which in both cases reflected a significant change (*p* < 0.05). Similar trends were observed for both the weekly numbers of prescribed and dispensed statin tablets per thousand of inhabitants, which increased within the analysed period from 652.4 to 1000.4, and from 445.1 to 817.1 tablets/week per 1000 inhabitants for prescribed and dispensed statins, respectively (*p* < 0.05). Figure 2 shows annual trends of weekly number of statin packs dispensed per 1000 citizens.

### 3.3 The percentage of dispensed statins

Despite these changes, the percentage of statin tablets dispensed was very stable. Linear regression proved a mean weekly increase of this parameter by 0.016%, leading to a statistically insignificant change of this percentage from 84.9% to 87.4% (*p* > 0.05) along the entire studied period. The mean percentage of statin tablets dispensed for every out of three studied years was in consequence very similar at approximately 86% (mean: 86.1 ± 4.9%, range: 85.9 ± 4.0% for 2022–86.4 ± 3.8% for 2021), and the observed differences were insignificant (*p* > 0.05).

**FIGURE 3:**
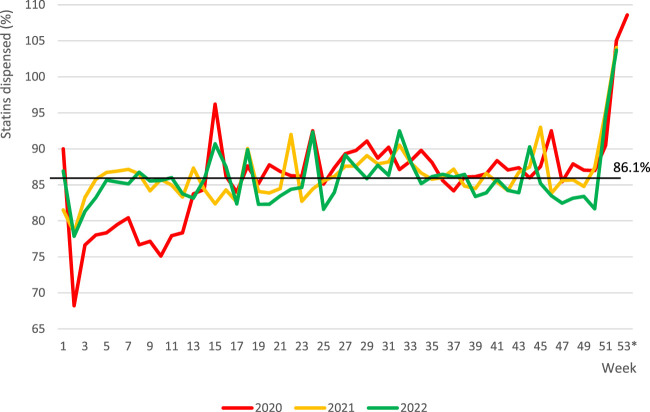
Fluctuations in the percentage of dispensed statins in Poland from 8 January 2020 to 31 December 2022. Note: horizontal line represents the mean value for entire period of analysis. *week 53 applicable to year 2020 only.

As illustrated in Figure 3, weekly fluctuation of the percentage of dispensed statin tablets showed clear repeatable characteristics. The highest values came with last weeks of the year (particularly in weeks 51–53), followed by a sharp drop in a few first weeks of the next year. More granular analysis revealed some additional information. For example, in the year 2020, there was a significant rise in week 14, preceding the Easter holiday, during the first lockdown, whereas a considerable decline was observed in week 18, which ended with the May long weekend (in Poland, 1st and 3rd of May are national holidays), preceded by an increase in week 17, etc. Data aggregated for trimesters proved some stable annual trends. The mean percentage ( ± SD) of statin tablets dispensed in the first trimesters of the years analysed (82.07 ± 3.82%) differed from those of the third and fourth trimesters (87.40 ± 1.49% and 87.52 ± 2.59%, respectively; *p* < 0.05).

### 3.4 Adherence and persistence

Overall adherence calculated for the entire analysis period was low: the mean MPR was 70.2 ± 37.3%. The percentage of adherent patients was low at 48.2% (and even lower at 38.7%, if the threshold of possession of ≥ 90% of doses was used).

**FIGURE 4:**
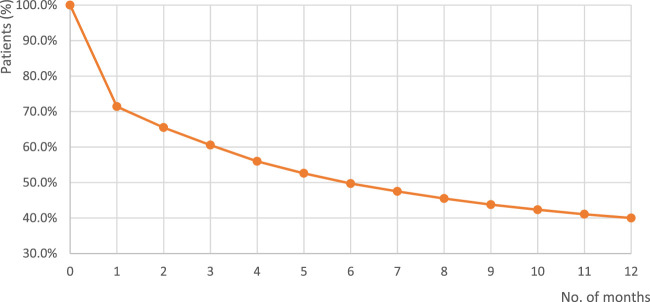
Kaplan-Meier curve of persistence with statin therapy of a random group of 100,000 Polish patients. Note: The time covered by this analysis was 1 January–31 December 2022; Base: a randomly selected cohort of 100,000 individuals who were statin users before 1 January 2022.

For a random sample of 100,000 patients, who were statin users before 1 January 2022, persistence was assessed for the period between 1 January and 31 December 2022. Figure 4 presents a Kaplan-Meier curve of persistence to statin therapy in that sample, and shows that at month 12 persistence was 40.0%, with the mean period of continuous therapy of 6.2 ± 5.3 months.

### 3.5 Effect of the COVID-19 pandemic and the related lockdown

**FIGURE 5:**
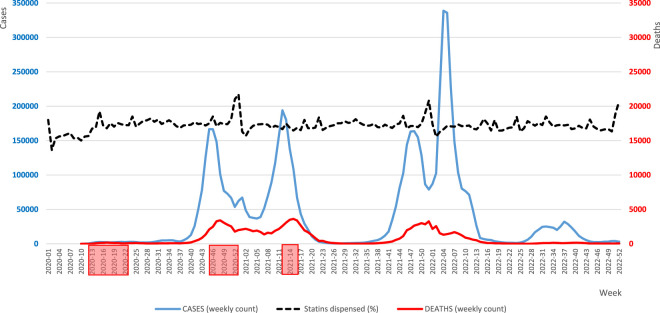
Weekly numbers of COVID-19 cases, COVID-19-related deaths and the percentage rates of statins dispensed in Poland during the pandemic period. Note: The analysed period spans from 8 January 2020 to 31 December 2022. The dotted line, illustrating the percentage of dispensed statins, is for illustrative purposes only and does not come with a scale. Lockdowns are indicated by red boxes along the timeline.

Figure 5 illustrates weekly numbers of COVID-19 cases, COVID-19-related deaths and the percentages of statins dispensed in Poland during the period of pandemic. The correlation analysis revealed a statistically significant but weak positive relationship between the number of COVID-19 cases and the quantity of statin tablets dispensed (*p* < 0.05). Similar correlation was observed for the number of statin tablets prescribed, however it was insignificant. No correlation was observed between weekly COVID-19 death counts and the number of prescribed or dispensed statins. Similarly, the percentage of dispensed statins showed no correlation with either the number of COVID-19 cases or deaths.

As regards the non-lockdown period, the correlation analysis demonstrated a statistically significant (*p* < 0.05) weak positive relationship between the number of COVID-19 cases and the quantity of dispensed tablets, however, not for prescribed tablets. Nevertheless, during the lockdown period, the mean number of prescribed and dispensed tablets was lower by 6.8% and 5.9%, respectively, compared to the non-lockdown period (in both cases, the differences being statistically significant, *p* < 0.05). On the other hand, the mean percentage of tablets dispensed during the lockdown period was slightly higher than that during the non-lockdown period (median ± SD: 86.3 ± 5.3% and 85.8 ± 4.9%, respectively, *p* > 0.05). A comparison of the weeks in 2020 and 2021 in which there were lockdowns with their corresponding “open” weeks in 2022 revealed that the percentage of dispensed tablets was higher during both the first and the second lockdown periods than outside them. In contrast, during the third lockdown period, the mean percentage of dispensed tablets was lower than outside it. However, in all these three cases, no statistically significant differences were found (*p* > 0.05).

## 4 Discussion

Due to high prevalence of ASCVD in Poland, statins play a pivotal role in public health. Good access to these drugs, and their continuous use by patients is of utmost importance for both primary and secondary prevention. This is particularly true given the rapidly aging Polish population, a factor that could further contribute to the increased incidence of ASCVD. Unfortunately, the recent outbreak of the COVID-19 pandemic created unfavourable conditions for maintenance of long-term therapies (Ágh et al., 2021; Kardas et al., 2021). Hence, as the first study of its kind to investigate statin use in Poland during the pandemic, this research yields doubly interesting results. It not only illustrates general aspects of prescribing, dispensing, adherence, and persistence with these drugs but also explores potential correlations with COVID-19 statistics. In fact, this research presents a comprehensive analysis of real-world data on a drug class of paramount importance. Using a nationwide database encompassing 38 million Polish citizens, the study provides robust evidence that will inform future preventive and corrective interventions.

An interesting observation of our study pertains to the consistent annual fluctuations in prescribing and dispensing of statins. The remarkable repeatability of trends in specific years is noteworthy. Towards the end of the year, there is a substantial drop in the number of both prescribed and dispensed tablets, which lasts till the first weeks of the new year. Similar observations have been described in another study performed during the COVID-19 pandemic in Italy (Olmastroni et al., 2020). It reflects the typical trend among patients who devoted less attention to management of their chronic asymptomatic conditions in the last days of the year and the first days of the new year. It is highly likely that this phenomenon was also related to the limited access to healthcare providers at that time. In the Italian study, a remarkable drop in dispensed statins was observed in August, which most probably corresponded to the traditional summer holiday period in this country. In Poland, this trend was not observed, however, altered dispensation was identified in other periods, i.e., various holidays such as Easter and the May long weekend.

Interestingly, there were distinct annual fluctuations in the percentage of dispensed tablets. The most notable variation, marked by peak values, occurred in the last weeks of the year, specifically in weeks 51–53, followed by a sharp decline in the initial weeks of the following year. These trends can be ascribed to the uncertainty surrounding the availability and pricing of medications in the coming year, combined with a potential hesitation to leave health issues unresolved from the previous year that was observed among those who had committed to therapy.

Despite these fluctuations, a continuous rising trend in both prescribing and dispensing was identified. To some extent, it could be explained by the demographics of the Polish population, being slowly modified by the aging process. However, it does not fully justify a nearly 20% rise in the quantity of statin tablets prescribed and dispensed within 3-year period covered by our analysis. Perhaps, it rather reflects better saturation of the national population with lipid-lowering therapies, occurring despite the unfavourable COVID-19 conditions.

In spite of the overall rise in prescribing and dispensing, the percentage of dispensed statins was very stable in our analysis. Throughout the entire 3-year period of the analysis, it approximated 86%. When interpreting this figure, it should be noticed that a small portion of these prescriptions were those issued for the first time to patients supposed to initiate statin treatment, whereas a majority were prescribed to the established users. Therefore, it is striking that in our nationwide cohort, every seventh prescription for statin was never filled in. Our previous study based on the data coming from the pilot implementation of e-prescriptions in Poland, which took place in 2018, demonstrated an even higher overall level of non-dispensation (20.8%), with prescriptions for statins not dispensed in 17.9% of cases (Kardas et al., 2020). This result is similar to that observed in the current study on the COVID-19 pandemic. Thus, the problem seems to be persistent, and definitely not related to the pandemic only. Of interest is the fact that in a 2021 analysis including six classes of drugs, performed in the neighbouring country of the Czech Republic (Bruthans et al., 2023), primary non-adherence was most prevalent (5.7%) for lipid-lowering drugs, underscoring statins as particularly prone to poor adherence. Indeed, our results coming from the analysis of real-world data of national cohort prove that non-adherence to statins is prevalent in Poland. In our study, the overall adherence calculated for the entire analysis period was low at MPR = 70.2 ± 37.3%, despite the fact that a large part of analysed patients entered the cohort over the way, i.e., was followed for a much shorter time than 3 years. Moreover, only 48.2% of these patients satisfied typical definition of adherence (i.e., MPR ≥ 80%). All of this must be interpreted in the context of the structure of the Polish healthcare system, which is universal and provides access to essential medications like statins with minimal co-payments (typically ranging from 1 to 5 euros per pack) or free of charge for individuals aged 75 and older.

Suboptimal adherence can diminish the potential benefits of lipid-lowering therapies, and lead to substantial increase in healthcare expenditures. Nevertheless, studies uniformly report alarmingly high levels of non-adherence with statins, in some cases reaching even higher than those observed in the present study. Various deviations from advised therapy may stand behind this problem. A study in older adults identified 4 distinct trajectories of statin use, out of which 3 accounted for non-adherence: gradual decline (16.8%), gaps in adherence (17.2%), and rapid discontinuation (7.8%), with only 58.2% of patients presenting high or nearly perfect adherence (Vadhariya et al., 2019). Not surprisingly, a recent meta-analysis proved suboptimal lipid management across Europe, and found adherence to lipid-lowering therapies to range between 46% and 92% (Barrios et al., 2021). In patients discharged with acute coronary syndromes in China, 72% adhered to statin therapy after 6 months only (Xie et al., 2017). Studies assessing patients newly prescribed statins found adherence to be extremely low in some cases, with 27% of men and 19% of women being adherent at 1-year only (Olmastroni et al., 2020; Ofori-Asenso et al., 2018a). Studies assessing adherence in a longer perspective provide equally striking results. A meta-analysis of data of more than 3 million older statin users in 82 studies proved that at 1-year follow-up, 59.7% (primary prevention 47.9%; secondary prevention 62.3%) of users were adherent, whereas at 3 and ≥10 years, 55.3% and 28.4% of users were adherent, respectively (Ofori-Asenso et al., 2018a). Recent studies echo comparable findings: the mean proportion of days covered (PDC) with statins at the conclusion of a 3.75-year period was 84% in Germany (Koenig et al., 2023). In Australia, the proportion of adherent individuals was 51% after 5 years (Talic et al., 2022), while in Taiwan, among patients who initiated statins post-hospital discharge for new-onset ASCVD, only 42% demonstrated adherence at the end of the seventh year (Chen et al., 2019).

It is noteworthy, however, that the studies cited above presented statistics on statin use from the pre-pandemic era. In contrast, our study, spanning the COVID-19 pandemic period, offers additional insights into statin use amid challenging conditions. It reveals that during the pandemic, Polish patients exhibited adherence and persistence levels comparable to those observed in studies conducted in other countries, outside pandemic scenarios.

In our study, persistence at month 12 was 40.0%, with the mean period of continuous therapy of 6.16 ± 5.31 months within the analysed period of 12 months. These results were very similar to those obtained in a study assessing a wide cohort (N = 613,654 patients) of new statin users prior to the COVID-19 pandemic, in which the percentage of persistent patients was 23.1% among men, and 15.8% among women, if the threshold of 30 days was used (Olmastroni et al., 2020).

Persistence with statins is generally very poor. Multiple studies from various locations report high percentage of new statin users who discontinue their treatment even within the first year after initiation, from 23.9%, on average, in meta-analysis of 82 studies (Ofori-Asenso et al., 2018a) up to 39% in Japan (Tomida et al., 2023), and 44.7% in Australia in recent reports (Ofori-Asenso et al., 2018b). Among patients with high-risk of CV events who newly initiated statin therapy in USA, median time to statin discontinuation was approximately 15 months (Lin et al., 2016). Among Scottish patients who initiated statin use for the secondary CVD prevention, 12% discontinued it within 1.5 years since initiation, and 19% within 3.5 years (Thalmann et al., 2023). In a longitudinal nationwide study assessing long-term persistence with statin therapy in Finland, only 43.9% were using statins up to the end of the 10th year of observation (Helin-Salmivaara et al., 2008). In a recent German analysis of lipid-lowering drugs use, at 12, 24 and 36 months after initiation the level of discontinuation was 60.8%, 73.8% and 79.4%, respectively (Lin et al., 2016). Interestingly, discontinuation is not limited to new users. Quite the contrary, in a nationwide study conducted among elderly Danes, approximately 19% of long-term statin users discontinued therapy within 2 years (Morotti et al., 2019).

On the other hand, an unexpected finding of this study is that the pandemic neither stopped new patients from initiating their statin therapy, nor the other patients from continuing it. Just the contrary, new individuals continuously initiated statin therapy, and the overall number of statin tablets prescribed and dispensed over the analysis period was continuously rising (see Figure 1). Indirectly, it confirms good access to medical services during the COVID-19 pandemic in Poland. Interestingly, the pandemic in a sense promoted statin use, as its exacerbation had a positive effect on the statin use: the higher the number of COVID-19 cases was, the higher were the numbers of statins prescribed (NS) and dispensed (*p* < 0.05). Although during the lockdown period, the mean number of prescribed and dispensed tablets was lower by 6.8% and 5.9%, respectively, as compared to non-lockdown periods (*p* < 0.05), the percentage of dispensed tablets showed the opposite, yet a non-significant tendency.

When interpreting these results, it should be emphasised that in Poland prescription drugs cannot be ordered in online pharmacies, and home delivery of such medications is not available. Such limitations created objective barriers to adherence at the pandemic peak, and particularly during lockdowns. However, our data prove that despite expectations, these factors did not constitute major obstacles to adherence during the COVID-19 pandemic.

The overall effect of the COVID-19 pandemic on adherence to chronic therapies seems to be ambiguous. As illustrated by the systematic review, a lot of chronic treatments were interrupted or negatively affected by the pandemic, due to various factors, such as fear of infection, lowered access to healthcare facilities, and unavailability of medicines. However, other therapies remained relatively unaffected, primarily because of the increased use of e-health tools and telemedicine (Olmastroni et al., 2020). Analysis of a large pharmacy claims database (over 250 million patients) found that most of American patients were able to access chronic medications in the early months of the COVID-19 pandemic, yet they were still more likely to discontinue their therapies than in the previous months. Moreover, at the time of the pandemic, there were fewer new patients who started taking their chronic medications (Clement et al., 2021). A study performed in Uganda found that risk of running out of antiretroviral drugs among HIV patients increased from 5% before the lockdown to 25% (Wagner et al., 2021).

Studies that looked specifically at adherence to stains during the pandemic yielded equally equivocal results. A few of these studies have been performed in Italy, a country heavily affected by the first wave of COVID-19. When comparing 2020 to the previous year, one study found that in the Pescara region, the adherence did not change much, unlike the persistence which dropped significantly (Romagnoli et al., 2022). Another study conducted in Lombardy identified an increase in PDC in March and April 2020, with a sharp decrease in May and June 2020. However, only a negligible decrease (−2.22%) in the total quantity of packs of lipid-lowering drugs dispensed in 2020 was observed (Casula et al., 2022). Another study found just a small rise in the proportion of failed refills of lipid-lowering drugs in April and May 2020 (42.4% and 42.5%, respectively *versus* 38.6% in the pre-COVID-19 period) (Degli Esposti et al., 2020). A Japanese study addressing the same issue identified no clinically meaningful difference in PDC between periods before and during the pandemic, despite a temporary decline in physician visits (Osawa et al., 2021). Finally, a study performed in Malesia observed a positive effect of lockdown on medication adherence with statins, which could be related to relaxed restrictions on medication prescriptions and larger quantities of medications supplied to patients due to the COVID lockdown, as well as the important role that telemedicine and mail-order pharmacies played during the pandemic period (Sim et al., 2023).

Finally, it needs to be acknowledged that this study has several limitations. Firstly, due to the use of prescription and dispensation data, the study could not assess the extent to which prescribed medications were actually used by patients. It was not possible to monitor daily variations of patient adherence to treatment either. Secondly, we do not have information regarding the extent of prescriber-initiated discontinuation of statins, discontinuation related to adverse effects, etc. Additionally, this study was not focused on factors affecting adherence. Some studies found adherence and persistence to statins to be related to patient and therapy characteristics, e.g., persistence was significantly higher in men than in women (Olmastroni et al., 2020), those taking higher number of prescribed medications (Morotti et al., 2019), and in patients prescribed high-intensity statin therapies (Rezende Macedo do Nascimento et al., 2020; Svensson et al., 2022; Koenig et al., 2023). What also matters are economic parameters, e.g., adherence to preventive statin therapy dropped with decreasing income (Wallach-Kildemoes et al., 2013); whereas increased co-payment either led to reduced use of statins, or their discontinuation (Seaman et al., 2021). A higher percentage of adherent patients was observed among users of generic statins vs. brand-name drugs (Gao et al., 2021). Moreover, when interpreting results of studies on adherence and persistence, one needs to consider various thresholds of discontinuation applied by their authors, ranging from 30 up to 270 days even (Helin-Salmivaara et al., 2008; Morotti et al., 2019; Ofori-Asenso et al., 2018a). This factor may have profound consequences for study results. For example, in one study 73.3% of the initiators continued statin therapy at the end of the first year with a 270-day gap between prescriptions used as a cut-off, whereas the proportions for 180-day and 90-day gaps were 69.0% and 56.7%, respectively (Helin-Salmivaara et al., 2008).

Nevertheless, our study has various strengths. First of all, the research was conducted on a large national database including 38 million citizens, which was feasible due to the electronic prescribing system introduced on the national level just before the outbreak of the COVID-19 pandemic. Moreover, national prescription and dispensation database offers precise and reliable data as, serving as the basis for reimbursement for pharmacies, they are carefully revived.

Results of this study indicate an urgent need to improve medication adherence to statins since these are drugs of the utmost importance for public health. Several interventions of proven effectiveness are available to change this scenario (Reston et al., 2020; Krüger et al., 2018). Unfortunately, current use of such adherence-enhancing interventions is more than limited, not only in Poland. Systematic search across Europe identified 13 reimbursed interventions in nine countries only (Ágh et al., 2021). Therefore, it is strongly recommended to enhance the implementation of initiatives and tools that support patients in regular drug intake. This recommendation is primarily directed towards the national regulator and payer organization, the National Health Fund. In particular, offering relevant support to prescribers, such as automated digital alerts facilitating timely prescribing of refills, appears to be an effective solution that may ensure unbroken continuity of therapy.

## 5 Conclusion

To the best of our knowledge, this is the first study focused on examining real-world adherence and persistence among Polish patients during the pandemic. According to its results, over the 3 years of the COVID-19 pandemic in Poland, there was a continuous increase in the number of statin tablets prescribed and dispensed. This suggests that, despite the potential limitations posed by the pandemic, access to statins remained easy, which may be attributed to the mass-scale implementation of the e-prescription system that became the compulsory prescribing mode at the beginning of 2020. However, it is crucial to realise that approximately one-seventh of prescribed statin doses were never dispensed, and the overall levels of adherence and persistence were low. This underscores the necessity for concerted efforts to change this scenario in Poland.

## Data Availability

The data analyzed in this study is subject to the following licenses/restrictions: Dataset belongs to public payer organisation. Requests to access these datasets should be directed to przemyslaw.kardas@umed.lodz.pl.
